# William H. Treat

**Published:** 1902-03

**Authors:** 


William H. Treat, son of E. B. Treat, and vice-president and treasurer of the publishing house of E. B. Treat & Co., died at his home at Mount Vernon, N.Y., December 27, 1901, aged 33 years. He was a business man of prominence and well known to the medical profession.

